# Feasibility of cardiovascular magnetic resonance derived coronary wave intensity analysis

**DOI:** 10.1186/s12968-016-0312-8

**Published:** 2016-12-09

**Authors:** Claire E. Raphael, Jennifer Keegan, Kim H. Parker, Robin Simpson, Julian Collinson, Vass Vassiliou, Ricardo Wage, Peter Drivas, Stephen Strain, Robert Cooper, Ranil de Silva, Rod H. Stables, Carlo Di Mario, Michael Frenneaux, Dudley J. Pennell, Justin E. Davies, Alun D. Hughes, David Firmin, Sanjay K. Prasad

**Affiliations:** 1 NIHR Cardiovascular Biomedical Research Unit, Royal Brompton & Harefield NHS Foundation Trust, London, UK; 2 Department of Bioengineering, Imperial College, London, UK; 3 Liverpool Heart and Chest Hospital, Imperial College Medical School, Liverpool, UK; 4 Norwich Medical School, University of East Anglia, Norwich, UK; 5 International Center for Circulatory Health, Imperial College, London, UK; 6 Institute of Cardiovascular Science, Faculty of Population Health Sciences, University College London, London, UK; 7 Department of Cardiovascular Magnetic Resonance, Royal Brompton Hospital, Sydney Street, London, SW3 6NP UK

## Abstract

**Background:**

Wave intensity analysis (WIA) of the coronary arteries allows description of the predominant mechanisms influencing coronary flow over the cardiac cycle. The data are traditionally derived from pressure and velocity changes measured invasively in the coronary artery. Cardiovascular magnetic resonance (CMR) allows measurement of coronary velocities using phase velocity mapping and derivation of central aortic pressure from aortic distension. We assessed the feasibility of WIA of the coronary arteries using CMR and compared this to invasive data.

**Methods:**

CMR scans were undertaken in a serial cohort of patients who had undergone invasive WIA. Velocity maps were acquired in the proximal left anterior descending and proximal right coronary artery using a retrospectively-gated breath-hold spiral phase velocity mapping sequence with high temporal resolution (19 ms). A breath-hold segmented gradient echo sequence was used to acquire through-plane cross sectional area changes in the proximal ascending aorta which were used as a surrogate of an aortic pressure waveform after calibration with brachial blood pressure measured with a sphygmomanometer. CMR-derived aortic pressures and CMR-measured velocities were used to derive wave intensity. The CMR-derived wave intensities were compared to invasive data in 12 coronary arteries (8 left, 4 right). Waves were presented as absolute values and as a % of total wave intensity. Intra-study reproducibility of invasive and non-invasive WIA was assessed using Bland-Altman analysis and the intraclass correlation coefficient (ICC).

**Results:**

The combination of the CMR-derived pressure and velocity data produced the expected pattern of forward and backward compression and expansion waves. The intra-study reproducibility of the CMR derived wave intensities as a % of the total wave intensity (mean ± standard deviation of differences) was 0.0 ± 6.8%, ICC = 0.91. Intra-study reproducibility for the corresponding invasive data was 0.0 ± 4.4%, ICC = 0.96. The invasive and CMR studies showed reasonable correlation (*r =* 0.73) with a mean difference of 0.0 ± 11.5%.

**Conclusion:**

This proof of concept study demonstrated that CMR may be used to perform coronary WIA non-invasively with reasonable reproducibility compared to invasive WIA. The technique potentially allows WIA to be performed in a wider range of patients and pathologies than those who can be studied invasively.

**Electronic supplementary material:**

The online version of this article (doi:10.1186/s12968-016-0312-8) contains supplementary material, which is available to authorized users.

## Background

Within the arterial system, waves are generated proximally by the contraction and relaxation of the myocardium during ejection. In the coronary arteries, there are also distally generated waves resulting from the compression and decompression of the intra-myocardial blood vessels which make interpretation of coronary artery wave mechanics much more difficult. Wave intensity analysis (WIA) allows phasic flow to be divided into a series of wavefronts that underlie the changes in pressure and flow seen within a vessel. The wave intensity is a measure of the power per unit area carried by the waves and its sign indicates the direction in which the waves are travelling; positive for forward and negative for backward travelling waves. These waves can be further classified into compression and expansion (decompression) based on their effect on pressure and acceleration and deceleration based on their effect on flow velocity.

**Fig. 1:**
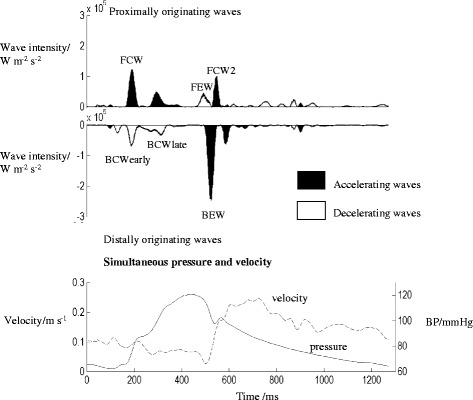
Wave intensity analysis in the healthy coronary circulation. A pattern of 6 waves is seen in the healthy circulation. Each acts to accelerate or decelerate the flow of blood in the epicardial coronary arteries. The top panel shows the WIA pattern. Proximally originating waves are displayed above the axis and distally originating waves below the axis. The bottom panel shows the pressure (solid line) and flow velocity (dashed line)

In the healthy heart there are 6 major waves that influence coronary flow, with the majority of coronary flow occurring in diastole due to a backward-travelling expansion wave due to decompression of the coronary microcirculation (Fig. 1). Studies in conditions with increased microvascular compression such as left ventricular hypertrophy [1] and aortic stenosis [2] have demonstrated attenuation of this wave, providing a mechanism for chest pain in these patients.

While invasive measurement has traditionally been used to acquire data for coronary WIA, this has several limitations, including the potential for complications resulting from coronary angiography and requirement for ionising radiation. This has largely limited wave intensity to a research procedure during clinically indicated cardiac catheterisation. Therefore patient selection is skewed by clinical requirement for cardiac catheterisation, truly asymptomatic patients are unlikely to be studied and serial assessment of wave intensity over time is challenging. A non-invasive method of coronary WIA is therefore desirable.

Non-invasive wave intensity requires measurement of velocity and pressure with high temporal resolution. For the coronary arteries, velocity data has been successfully measured using ultrasound (echocardiography) [3, 4] while distension of the carotid arteries calibrated to brachial blood pressure has been used as a surrogate measure for the arterial pressure waveform [5, 6], However, visualisation of the coronary arteries using echocardiography can be challenging and is usually limited to the mid-distal LAD. Measurement is complicated by off-axis imaging planes [3]. Cardiovascular magnetic resonance (CMR) offers several potential advantages in flow velocity measurement. While underestimating absolute measures of coronary flow velocity, high temporal resolution (19 ms) interleaved spiral phase velocity using CMR has recently been shown to allow accurate and reproducible assessment of the temporal patterns of coronary artery blood flow [6]. Using this technique, through-plane velocity data with high sensitivity to flow can be acquired in a breath-hold, with retrospective ECG gating allowing full coverage of the cardiac cycle in typically 50 frames. Although, as expected, measured velocities are under-estimated relative to invasive Doppler guide wire due to partial volume averaging, temporal flow patterns in both the left and right coronary arteries have been shown to agree well with those seen invasively [7].

CMR measurement of aortic distension has been shown to provide a good estimate of central aortic pressure [8] and central aortic pressure provides a suitable surrogate for coronary pressure [6]. WIA has recently been successfully performed using CMR in the ascending and descending aorta [9], however it has never been attempted in the highly-mobile, small-caliber, tortuous coronary arteries, nor has it been directly compared to invasively acquired data. Accordingly, we assessed the feasibility of coronary WIA using CMR and compared to invasive measures in the same patients.

## Methods

CMR scans were undertaken in a serial cohort of patients who had undergone invasive WIA (8 patients, 12 coronary arteries). Exclusion criteria were inability to undergo CMR, valvular heart disease and coronary artery disease with 50% or greater luminal stenosis in any epicardial vessel greater than 2 mm. Patients were recruited from the Royal Brompton and Harefield NHS Foundation Trust and Liverpool Heart and Chest Hospital NHS Foundation Trust. All patients gave written consent for additional research measurements. The study was approved by an independent ethics committee.

### Invasive data acquisition

Following coronary angiography, if no significant epicardial coronary artery disease was identified, a Doppler velocity and pressure wire (Combowire, Volcano Therapeutic) was positioned in the proximal left anterior descending (LAD) and proximal right coronary artery (RCA). Wire position and signal were optimised and simultaneous recordings of pressure, flow velocity and electrocardiogram were acquired at 200Hz, for a period of 60 s. To assess reproducibility, a further recording was taken in the same vessel location.

### Acquisition of CMR data

**Fig. 2:**
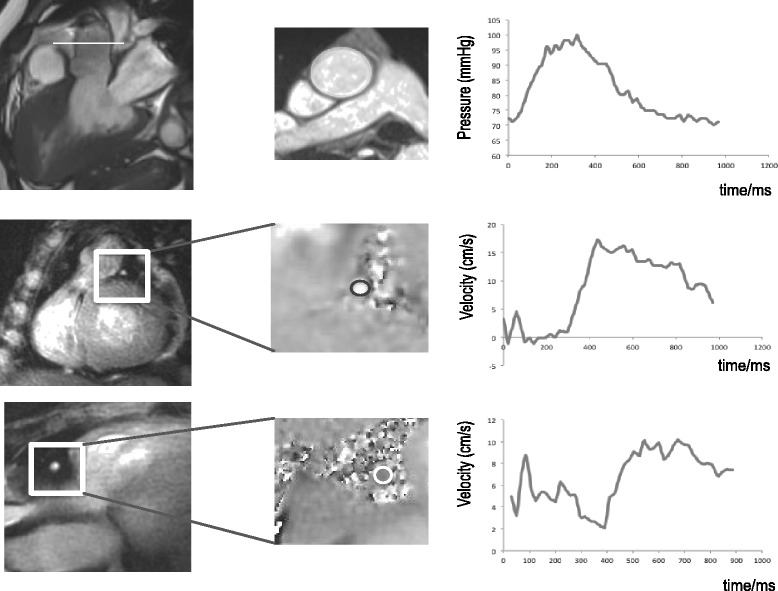
For derivation of pressure data (top panel), a cross sectional plane was identified 35 mm above the aortic valve plane. A high temporal resolution gradient echo sequence was used to acquire aortic areas throughout the cardiac cycle and these were used to derive the central aortic pressure during the cardiac cycle. Early diastolic cross sectional imaging of LAD (middle panel) and RCA (bottom panel) using breath-hold spiral phase velocity mapping

CMR images were acquired using a 3 Tesla Magnetom Skyra scanner (Siemens AG Healthcare Sector, Germany). Following acquisition of localiser images and vertical and horizontal long axis balanced steady state free precession (bSSFP) cine images of the heart, bSSFP cine images were acquired in the three chamber and left ventricular outflow tract views to show the ascending aorta in-plane. A breath-hold retrospectively gated segmented gradient echo sequence (TE/TR 3.6 ms/6.6 ms) was then used to acquire through-plane cross sectional area changes in the proximal ascending aorta. The spatial resolution was 1.2 × 1.2 mm (reconstructed to 0.6 × 0.6 mm) with a slice thickness of 8 mm. Five k-space lines were acquired per cardiac cycle resulting in data with a true temporal resolution of 32.8 ms which was then reconstructed over 50 cine frames (reconstructed temporal resolution 20 ms assuming a heart rate of 60 beats per minute)(Fig. 2, top panel). The acquisition plane was located 35 mm above the aortic root as this was typically in a straight section of the aorta with minimal through-plane motion through the cardiac cycle. The segmented gradient echo cine acquisition was repeated for assessment of intra-study reproducibility. In each, the aortic cross sectional area was manually contoured and plotted against time from the R wave.

The coronary artery origins were identified using multiple transverse diastolic segmented gradient echo scout acquisitions (TE/TR: 3.3 ms/7 ms, acquired resolution 1 mm × 1 mm × 4 mm, acquisition window 110 ms). From these, oblique and double oblique images were acquired to show the left anterior descending (LAD) and right coronary artery (RCA) in-plane. Through-plane breath-hold interleaved spiral phase velocity maps (TE/TR: 5.2 ms/19 ms) were then acquired in a straight section of each proximal artery, matched as closely as possible to the locations of the invasive measurements (Fig. 2, mid and lower panels). The sequence incorporated 1–1 water excitation and 8 spiral interleaves (11.75 ms duration) were required to fill k-space. Phase map subtraction of datasets with symmetric bi-polar velocity encoding gradients resulted in through-plane velocity maps sensitive to a flow velocity of ±30 cm/s. These datasets were acquired in alternate cardiac cycles following a single dummy cardiac cycle, resulting in a total breath-hold duration of 17 cardiac cycles. The spatial resolution was 1.4 mm × 1.4 mm (reconstructed to 0.7 mm × 0.7 mm) and the slice thickness was 8 mm. Locations of velocity measurement were directed by the invasive fluoroscopic images to ensure the same location as invasive data acquisition where possible. Sensitivity to off-resonance artefact was minimised using localised second-order shimming and frequency adjustment based on the signal from a user-defined region of interest positioned over the heart. For right coronary studies, an additional breath-hold spiral phase velocity mapping acquisition was performed using fat-excitation [10]. This was later used to correct for the through-plane velocity of the RCA.

For each breath-hold acquisition, following multi-level thresh-holding [11], a circular cross-sectional area was automatically defined over the vessel on a mid-diastolic frame using a modified Hough-based transform [12]. Semi-automatic custom MATLAB software was then used to track the artery from frame to frame and to generate a velocity-time curve [7]. As the spatial resolution of the data was limited, no attempt was made to measure changes in the vessel cross-sectional area throughout the cardiac cycle. The velocity-time curves were corrected for through-plane motion of the vessel using a region of adjacent myocardium (LAD) or surrounding epicardial fat (RCA) [7, 10]. The velocity-time curves obtained with this approach have been shown to have a high inter breath-hold reproducibility and the temporal patterns measured are very similar to those obtained with invasive Doppler guidewire [7]. As expected, the CMR velocities are lower than those obtained invasively where the peak velocity is determined and while this will affect the derived absolute wave intensities, the proportional wave intensities – which are based on temporal patterns of velocity and pressure (rather than on absolute values)—should be unaffected.

To minimise circadian variation in coronary flow between the CMR and invasive studies, readings were taken under standardised conditions. Both procedures were performed on the same day if possible with 1 h between procedures. If CMR and invasive measurement were performed on separate days, they were performed at the same time of day for both studies. There was a minimum of 30 min lying supine at rest prior to data acquisition. Patients refrained from caffeine containing substances and smoking for at least 24 h prior to each procedure. There was a minimum of 30 min lying supine at rest prior to data acquisition. Patients refrained from caffeine containing substances and smoking for at least 24 h prior to each procedure. Prescribed medications were not changed between the two studies.

### Derivation of central aortic pressure from aortic distension data

The central aortic pressure was derived from the aortic distension calibrated to brachial blood pressure as previously described [8, 13]. This exponential model correlates well with the results of carotid artery applanation tonometry [8] which has been validated against invasive central aortic pressure measurements [14]. Subjects with maximal aortic distension of less than 10% were excluded from the study as area changes through the cardiac cycle were too small to be measured accurately.

### Wave intensity analysis of invasive data

Analysis of coronary wave intensities requires simultaneous assessment of the first derivatives of central aortic pressure and of coronary blood flow velocity, together with an estimate of the wave speed. Stepwise description of data processing for wave intensity analysis is shown in Additional file 1: Figure S1. The waterhammer equations allow description of wavefronts according to the changes in pressure and velocity. They are derived from conservation of mass and momentum. The water hammer equations are used to derive the forward and backward originating waves as follows [15, 16]:

Proximally originating wave intensity:

$$ W{I}_{+}=\frac{1}{4\rho c}{\left(\frac{dP}{dt}+\rho c\frac{dU}{dt}\right)}^2 $$

Distally originating wave intensity:

$$ W{I}_{-}=-\frac{1}{4\rho c}{\left(\frac{dP}{dt}-\rho c\frac{dU}{dt}\right)}^2 $$

Where ρ is the blood density (taken as 1050 kg/m^3^, c is the local wave speed, dU is the incremental change in coronary flow velocity and dP is the incremental change in coronary artery pressure and + and—indicate forward and backward waves.

The wave speed is calculated using the following formula (sum of squares method), where the sums are taken over the cardiac period [17]:

$$ c = \frac{1}{\rho\ }\sqrt{\frac{{\displaystyle \sum }d{P}^2}{{\displaystyle \sum }d{U}^2}} $$

Cumulative wave intensity of each wave was calculated as the area under each peak. For comparison between techniques and between patients, we presented the cumulative wave intensity of a particular separated wave as a proportion of the total cumulative wave intensity, expressed as a percentage.

### Wave intensity analysis of invasive and CMR data

For the invasive data, four to five beats of pressure and flow velocity data were ensemble averaged using the ECG R wave as the fiducial point. The mean aortic velocity and aortic-distension derived central aortic pressure were calculated as described above and linearly interpolated to the same sample frequency as the invasive data (200 Hz). Data from invasive and CMR methods were processed using a customised automated Matlab analysis program as previously described [1]. This used a Savitsky-Golay filter to smooth the data and estimate the derivatives [18] and the sum of squares method was used to derive the wave speed [19].

### Statistical analysis

All analyses were performed using IBM SPSS Statistics 19 Package. Normally distributed data are presented as mean ± standard deviation and categorical data as number (percentage of total). Non-normally distributed data are presented as median (interquartile range). The cumulative wave intensity of the principal waves through the cardiac cycle were calculated for each subject using invasive and CMR data as described above and the values derived from the two techniques compared using Bland-Altman analysis and Pearson correlation. For both invasive and non-invasive data, intra-study reproducibility of the proportional cumulative wave intensities was determined using Bland Altman analysis and reported as mean difference (±SD of the differences), and by calculation of the intraclass correlation coefficient (ICC) (two-way random effects model). The within-subject coefficient of variation was calculated as the within-subject standard deviation of the paired measurements divided by the mean of all measurements, expressed as a percentage. Agreement between the proportional cumulative wave intensities of the first invasive and first CMR measurements was assessed using Bland Altman analysis.

## Results

CMR and invasive catheter measurements were completed as specified in 8 subjects (12 coronary arteries—8 LAD, 4 RCA). Baseline characteristics for these patients are summarized in Table 1. Indication for cardiac catheterisation was hypertrophic cardiomyopathy (6 patients) or atypical chest pain (2 patients). Central blood pressures derived from CMR distension were higher than invasively measured values (SBP 130 ± 22 mmHg versus 115 ± 22 mmHg, *p <* 0.01) and heart rate was higher (71 ± 11 versus 66 ± 9 beats per minute, *p =* 0.19).

**Table 1:** Characteristics of all subjects

Age	49.8 ± 13.3
Male, n (%)	6 (75%)
Coronary flow reserve	1.7 ± 0.7
Co morbidities	
Hypertension	2 (25%)
Diabetes	0 (0%)
Hypercholesterolaemia	2 (25%)
Current Smoker	1 (13%)
Medications	
Beta blockers	5 (63%)
ACE inhibitors	0 (0%)
Calcium channel blockers	0 (0%)
Aspirin	2 (25%)
Statin	2 (25%)

### Agreement with invasive wave intensity analysis

Typically, wave intensity of the coronary arteries shows 6 dominant waves: the forward compression wave (FCW), forward expansion wave (FEW), second forward compression wave (FCW2), early backward compression wave (early BCW), late backward compression wave (late BCW) and backward expansion wave (BEW) (Fig. 1). However as most of our subjects had HCM, the early and late backward compression waves overlapped and were therefore measured as a single wave, the backward compression wave total (BCWtot).

Figue 3 shows CMR and invasive WIA in an example patient with CMR showing the expected pattern and relative intensity of the dominant waves (BCWtot, BEW and FCW). While the wave distribution profiles are similar, differences in the trigger times between the techniques resulted in consistently earlier timing of CMR waves compared to the invasive waves (Table 2). Summary values for invasive and CMR derived separated cumulative wave intensity values (both absolute and proportion) are presented in Table 3. The absolute values were higher for invasively derived data compared to CMR derived data as the invasive WIA uses the peak flow velocity within the vessel while with CMR the average flow velocity was measured across the vessel cross-section. Comparison of invasive and CMR-derived pressure and velocity data are shown in Additional file 2: Figure S2 and Additional file 3: Figure S3. The proportional wave intensities were similar for each technique although CMR slightly over-estimates BCWtot (41.5% vs 33.4%, *p <* .05). Figure 4 shows Bland Altman and linear regression plots for CMR versus invasive proportional wave intensities. The non-invasive and invasive studies showed reasonable agreement (*r =* 0.73, *p <* 0.001) with a mean difference (±SD) of 0.0 ± 11.5%. Additional file 4: Figure S4 and Additional file 5: Figure S5 show reproducibility and Bland-Altman plots for individual waves using invasive and CMR-derived wave intensity.

**Fig. 3:**
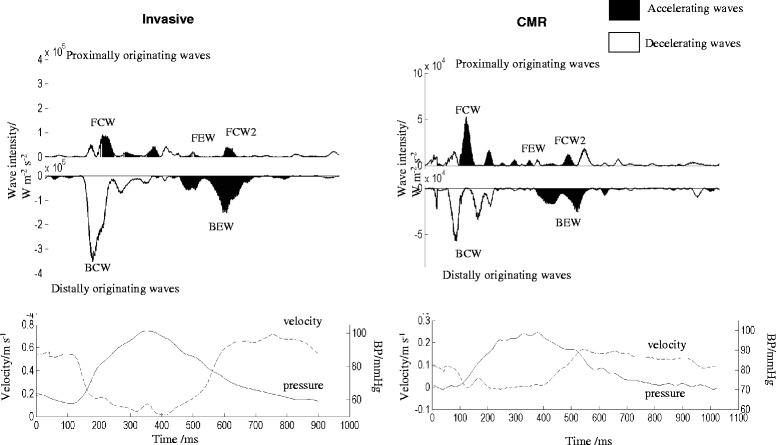
Visual comparison of WIA using invasive (left) and CMR derived (right) data in the same patient, with the corresponding pressure and flow velocity data shown below. While the absolute values for each wave were greater for invasive compared to CMR measures, the pattern of wave intensity and relative size of each wave was similar between the two traces

**Table 2:** Timing of waves using invasive and CMR measurements

	Onset of invasive wave peak/ms	Onset of CMR wave peak/ms	Difference between invasive and CMR peaks/ms
Forward compression wave	185 ± 21	116 ± 17	70 ± 28
Forward expansion wave	485 ± 41	414 ± 102	71 ± 105
Forward compression wave 2	540 ± 48	502 ± 92	37 ± 113
Backward compression wave	255 ± 54	197 ± 58	58 ± 80
Backward expansion wave	530 ± 33	471 ± 100	59 ± 111

**Table 3:** Invasive and CMR derived values for separated cumulative wave intensity analysis (absolute and proportion)

	Separated cumulative wave			Proportional separated cumulative wave intensity /%		
	intensity /Wm^−2^s^−1^ x 10^6^					
	Invasive	CMR	*p-*value	Invasive	CMR	*p-*value
Forward compression wave (FCW)	6.6 ± 4.3	4.1 ± 2.3	0.01	30.0 ± 15.4	24.4 ± 8.3	0.10
Forward expansion wave (FEW)	1.7 ± 3.2	1.2 ± 1.2	0.98	5.6 ± 5.4	6.7 ± 4.9	0.99
2nd forward compression wave (FCW2)	2.4 ± 3.3	2.5 ± 0.6	0.23	5.2 ± 3.0	4.2 ± 2.5	0.38
Backward compression wave (BCWtot)	8.7x ± 6.0	6.6 ± 3.4	<0.01	33.4 ± 11.0	41.5 ± 11.1	0.02
Backward expansion wave (BEW)	7.4 ± 5.7	4.1 ± 2.7	<0.01	25.7 ± 8.6	25.0 ± 6.3	0.85

Mean and SD are displayed with absolute WIA values (left half of table) and as a proportion of the total wave intensity (right half of table). The absolute values were higher for invasively derived data compared to CMR. When expressed as a % of total wave intensity, the BCW was greater with CMR but the other waves were similar

**Fig. 4:**
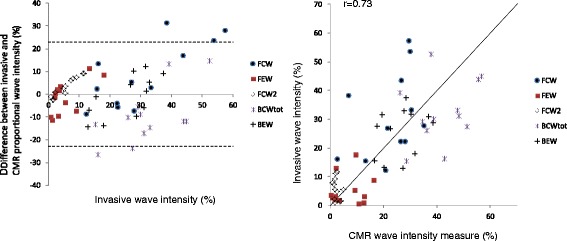
Comparison of invasive and CMR-derived proportional cumulative wave intensity analysis. All 5 waves are displayed on a single plot, assessed for agreement with invasive data as the gold standard using Bland Altman analysis (left panel, dotted lines are mean ± 2SD) and using the Pearson correlation coefficient (right panel)

**Fig. 5:**
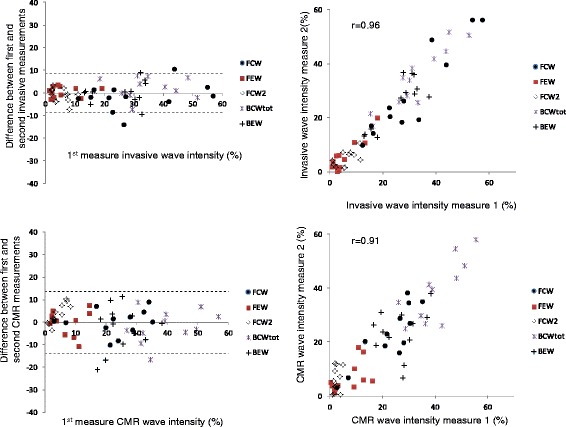
Reproducibility of invasive (top panels) and CMR-derived (bottom panels) wave intensity values using Bland Altman plots (left, dotted lines are mean ± 2SD) and Pearson correlation coefficient (right)

The intra-study reproducibilities of the proportional separated cumulative wave intensities by both invasive and CMR techniques are shown in the Bland Altman plots of Fig. 5, together with linear regression plots. The intra-study reproducibility of the CMR derived proportional separated cumulative wave intensities (mean ± standard deviation of differences) was 0.0 ± 6.8% with an ICC of 0.91 (95% confidence interval: 0.85 –0.95) and a co-efficient of variation of 23%. Intra-study reproducibility for the corresponding invasive data was 0.0 ± 4.4% with an ICC of 0.96 (95% confidence interval: 0.94 –0.98) and coefficient of variation 16%. The intra-study reproducibilities for each individual wave are shown in Table 4.

**Table 4:** Mean and standard deviation of the difference for invasive and CMR proportional cumulative wave intensities (%)

	Invasive vs Invasive data	CMR vs CMR data	Invasive vs CMR data
Forward compression wave (FCW)	−1.6 ± 6.2	0.2 ± 5.9	8.3 ± 14.7
Forward expansion wave (FEW)	0.4 ± 2.2	0.2 ± 4.8	−1.2 ± 7.6
Forward compression wave 2 (FCW2)	−1.1 ± 3.0	3.4 ± 4.4	3.3 ± 4.5
Backward compression wave total (BCWtot)	2.1 ± 4.7	−1.1 ± 7.3	−9.6 ± 13.1
Backward expansion wave (BEW)	0.1 ± 5.1	−2.8 ± 9.2	0.8 ± 9.5

Pearson correlation plots for individual waves (Additional file 4: Figure S4) show that BCW and FCW have the highest correlation between repeat measurements for both invasive (R 0.93 and 0.92 respectively) and CMR techniques (R 0.86 and 0.82 respectively), and also between CMR and invasive techniques (R 0.55 and 0.44 respectively). Individual wave Bland Altman plots for CMR reproducibility, invasive reproducibility and for a comparison between CMR and invasive techniques are presented in Additional file 5: Figure S5.

## Discussion

In this study, we demonstrated that CMR derived velocity and pressure data have the potential to be used for wave intensity analysis of the proximal coronary arteries. We performed CMR and invasive wave intensity analysis in 12 unobstructed coronary arteries and compared the absolute and percentage values of each wave using the two measurement techniques. CMR analysis produced similar patterns of wave intensity to invasive data and showed reasonable reproducibility compared to the invasive technique.

### The need for non-invasive coronary wave intensity analysis

Invasive wave intensity in the coronary arteries provided a definitive answer to why coronary flow peaks in diastole [1]. The complex interaction between the epicardial coronary arteries, ventricular contraction and relaxation and blood within the intra-myocardial vessels was elegantly described throughout the cardiac cycle through identification of six predominant waves that governed changes in coronary flow [20]. In the healthy heart, a large backward expansion wave was responsible for the increased coronary flow in early diastole, as compression on the intra-myocardial vessels is relieved [1]. Wave intensity has been used to explain the effect of mechanical treatments such as intra-aortic balloon pumps [21] and aortic valve replacement [2] on coronary filling patterns.

While the technique has provided valuable advances in understanding of the mechanisms responsible for abnormalities in coronary filling, expansion into a wider range of pathology has been limited by the requirement for invasive study. Currently, coronary wave intensity data has largely been derived from invasive measure, requiring coronary intubation and placement of a coronary wire into the artery for measurement of phasic pressure and flow changes. This has limited recruitment of patients for research which means that healthy controls have not been studied, nor the majority of coronary pathology.

Coronary flow abnormalities have been shown in a large population of cardiovascular disease [22–25]. Study with wave intensity is likely to allow better understanding of the mechanism of chest pain and perfusion abnormalities in these disease states. Study in larger patient cohorts and with longitudinal repeat measurement may also allow assessment of medication effects and even prognosis. A non-invasive technique for coronary wave intensity analysis is therefore desirable.

### CMR compared to echocardiography for non-invasive coronary artery velocity acquisition

Non-invasive wave intensity analysis in the coronary arteries was recently performed using echocardiography [6]. In that study, the cumulative separated backward expansion wave was similar to that from invasively acquired data while all other waves were underestimated. Echocardiography is limited by the need for favourable acoustic windows and the recorded coronary flow velocity will be highly dependent on the angle of the ultrasound beam in relation to the coronary artery [26]. A significant advantage of CMR is the ability for cross sectional imaging in any plane, ensuring that coronary flow is measured perpendicular to the vessel [27]. CMR will also allow measurement at any proximal location in the coronary tree and is not limited by vessel location relative to the ultrasound probe. Recent advances in CMR using a retrospectively gated flow velocity sequence allowed acquisition over the entire cardiac profile with a high temporal resolution sufficient for wave intensity analysis. The temporal patterns of velocity obtained with this technique in the coronary arteries have been shown to have a high reproducibility [7].

Biglino *et al.* performed wave intensity analysis using CMR in the ascending and descending aorta in healthy controls, patients with coronary heart disease [9] and congenital heart disease [28]. They demonstrated that wave intensity in the aorta was feasible using phase contrast CMR, with temporal wave intensity profiles that were in keeping with previously demonstrated invasive profiles, however they did not compare the technique with invasive data. In both their study and ours, spiral k-space trajectories allowed sufficient temporal resolution for velocity data, producing a suitable velocity profile for wave intensity analysis.

### Use of aortic distension as a surrogate measure for pressure

Vessel distension has been successfully employed as a surrogate for pressure changes within the vessel of interest in the ultrasound derived carotid [5, 29] and CMR derived aortic [9, 28] WIA papers. We elected to use changes in proximal aortic distension to derive changes in central aortic pressure over the cardiac cycle as this had previously been validated using CMR [8]. This assumes linearity between pressure and area [30]. Compared to vessel diameter changes, which was the technique employed for ultrasound-derived wave intensity in the carotid arteries, measurement of cross sectional area changes offered several advantages. Firstly, measurement error in calculation of vessel distension was likely to be smaller compared to calculation from aortic diameter, since areas changes derived from vessel diameter changes required squaring of the measurements, increasing the potential for error. Secondly, contouring of vessel area meant there was no assumption of vessel circularity throughout the cardiac cycle.

Aortic distension is known to vary between individuals. Older patients and those with longstanding hypertension typically have a reduction in aortic distension and increased stiffness of the aorta [31–33]. Patients with hypertrophic cardiomyopathy have also been shown to have reduced aortic distension [34]. In this feasibility study, we included only patients with an aortic distension of 10% or greater. Use of alternative non-invasive central aortic pressure surrogates such as a supra-systolic brachial cuff [35] or tonometry may allow study of patients with minimal aortic distension and would likely improve non-invasive wave intensity analysis techniques.

### Feasibility of CMR-derived WIA

This study is the first to successfully use CMR data to derive wave intensity of the proximal coronary arteries and showed acceptable agreement with invasive data. Compared to invasively derived WIA, CMR data showed greater standard deviation of the difference. The variability may be reduced by averaging the results of several breath-hold acquisitions. However, this would increase acquisition and analysis time, with potential for mis-registration between breath-holds.

A further challenge is that CMR measurement of coronary flow velocity requires data acquisition over several cardiac cycles. Variation in cardiac cycle length may affect wave intensity values, potentially more so during diastole and is a disadvantage of the CMR technique. Patients had a higher resting heart rate and blood pressure during the invasive data acquisition compared to CMR, so the pattern of waves governing coronary haemodynamics may not have been the same between the two techniques. Table 2 shows that on average, CMR waves were approximately 60 ms earlier than the corresponding invasive waves. This difference is reasonably consistent and is likely to be due to differences in the R-wave detection algorithms used in the invasive and non-invasive acquisitions. The greater spread in the timing of CMR wave peaks potentially results from the reduced temporal resolution of the non-invasive technique, or may be a result of R-R interval variability through the data acquisition. The latter would potentially broaden the peaks and make it more difficult to determine the peak position.

Bland Altman analysis showed that reproducibility of both techniques was acceptable although better for the invasive studies (SD of differences 4.4% compared to 6.8%). Analysis of within-subject CoV similarly showed better reproducibility of the invasive studies (16% compared to 23%) although it should be noted that both CoV values are inflated as the analyses are performed for the proportional wave intensities, rather than for the absolute values —since the proportional wave intensities in any subject must add to 100%, errors in any single wave automatically result in errors in the others (to maintain a sum of 100%) which leads to increased CoV values. ICC values were high for both invasive and CMR techniques (0.96 and 0.91 respectively).

The BCW and FCW had the highest correlation between repeat measurements for both invasive (R 0.93 and 0.92 respectively) and CMR techniques (R 0.86 and 0.82 respectively), and the highest correlation between invasive and CMR measures (R 0.55 and 0.44 respectively).

Currently, compared to CMR, invasive data acquisition is simpler and allows acquisition of multiple cardiac cycles, although it significantly limits the population likely to be studied. CMR WIA may allow longitudinal follow up of the same patient, to assess response to treatment and may allow derivation of normal ranges for individual waves in the entirely healthy population (Table 5). Further work to evaluate alternative pressure-surrogate measures and establish whether averaging across multiple velocity and pressure cycles derived from CMR results in improved reproducibility may further improve the technique.

**Table 5:** Comparison of invasive and CMR wave intensity analysis

	Advantages	Disadvantages
Invasive WIA	• More reproducible • More established evidence base in different pathologies	• Requirement for ionising radiation • Potential complications of invasive catheterisation • Difficult to measure bi-directional flow • Data quality very operator dependent
CMR WIA	• No ionising radiation • Easier to perform serial studies • Allows study of healthy population	• More time consuming post-processing • Requirement for pressure surrogate rather than direct pressure measurement • May not be valid in patients with increased aortic stiffness • Limited spatial resolution and partial volume averaging result in underestimation of flow velocities and absolute wave intensity peaks • Data acquired over multiple cardiac cycles

### Study limitations

This was a small feasibility study. In the CMR study, pressure and flow velocity data were not acquired simultaneously and the pressure data were derived from the proximal ascending aorta rather than the coronary artery. However, all data were gated to the R wave which enabled alignment of the velocity and derived pressure traces. We assumed linearity between pressure and area in the range of aortic distention measured in this study which was in keeping with previous work [8].

Due to the selection of our subjects, who largely had hypertrophic cardiomyopathy, reversal of coronary flow during systole was common. This produced limitations in the measurement of invasive data, as the internal algorithm of the Combowire struggled to identify small reversals in coronary flow and often instead traced the positive reflected “mirror” of the coronary flow trace. With CMR data, there was no mirroring and the technique clearly differentiated forward and reverse flow.

Where possible, CMR and invasive measures were obtained on the same day, however this was not always possible due to patient preference and work flow logistics. Measurements were taken for both CMR and invasive wave intensity analysis with patients supine and resting for at least half an hour before data acquisition, however the physical and physiological added stresses of the invasive procedure meant that resting blood pressure and heart rate were higher at the time of invasive measurement compared to CMR measurement.

As expected, CMR measured velocities were under-estimated relative to invasive Doppler guide wire due to partial volume averaging, leading to lower absolute values of WIA using CMR compared to invasive. Application of a Poiseuille or Womersley model to the invasive data would allow estimation of average flow velocities and potentially improve agreement between invasive and non-invasive estimates of flow but would require multiple assumptions that may not be valid in the coronary circulation. We have, instead, focussed on proportional cumulative wave intensities which require temporal patterns of velocity and pressure, rather than absolute values of both.

## Conclusion

CMR derived pressure and flow data can be used to perform coronary wave intensity analysis. The technique may not be suitable in older and hypertensive patients in whom increased aortic stiffness leads to lower aortic distension. However, in selected patients, there was acceptable agreement with invasive data and good reproducibility.

## Additional files

Additional file 1: Figure S1. Stepwise analysis of invasive and CMR data. Ad = diastolic aortic area, As = systolic aortic area, Ps = brachial systolic pressure, Pd = brachial diastolic pressure, ∝ = scaling factor. *ρ* = blood density (taken as 1050 kg/m^3^). (PPTX 103 kb)

Additional file 2: Figure S2. Comparison of invasive and CMR-derived pressure readings. (PPTX 205 kb)

Additional file 3: Figure S3. Comparison of invasive and CMR flow velocity readings. (PPTX 115 kb)

Additional file 4: Figure S4. Comparison of reproducibility of invasive and CMR data. The top row shows invasive vs invasive data, the middle row CMR vs CMR data and the bottom row invasive vs CMR data. (PPTX 164 kb)

Additional file 5: Figure S5. Bland Altman plots for reproducibility of individual waves using invasive (top row) and CMR (middle row) modalities. Comparison of individual waves using CMR compared to invasive data acquisition are presented in the bottom row. (PPTX 222 kb)

## Abbreviations

BCW: Backward compression wave
BEW: Backward expansion wave
CMR: Cardiovascular magnetic resonance
CoV: Coefficient of variation
ECG: Electrocardiogram
FCW: Forward compression wave
FCW2: Forward compression wave 2
FEW: Forward expansion wave
ICC: Intraclass correlation coefficient
LAD: Left anterior descending
RCA: Right coronary artery
WIA: Wave intensity analysis
