# Supplementary material for: Feasibility of cardiovascular magnetic resonance derived coronary wave intensity analysis
Source: J Cardiovasc Magn Reson. 2016 Dec 9;18:93. doi: 10.1186/s12968-016-0312-8 (PMC5154155; doi:10.1186/s12968-016-0312-8)
Supplement: Supplementary file 1 — Stepwise analysis of invasive and CMR data. Ad = diastolic aortic area, As = systolic aortic area, Ps = brachial systolic pressure, Pd = brachial diastolic pressure, ∝ = scaling factor. ρ = blood density (taken as 1050 kg/m3). (PPTX 103 kb) [file 12968_2016_312_MOESM1_ESM.pptx]

## Slide 1
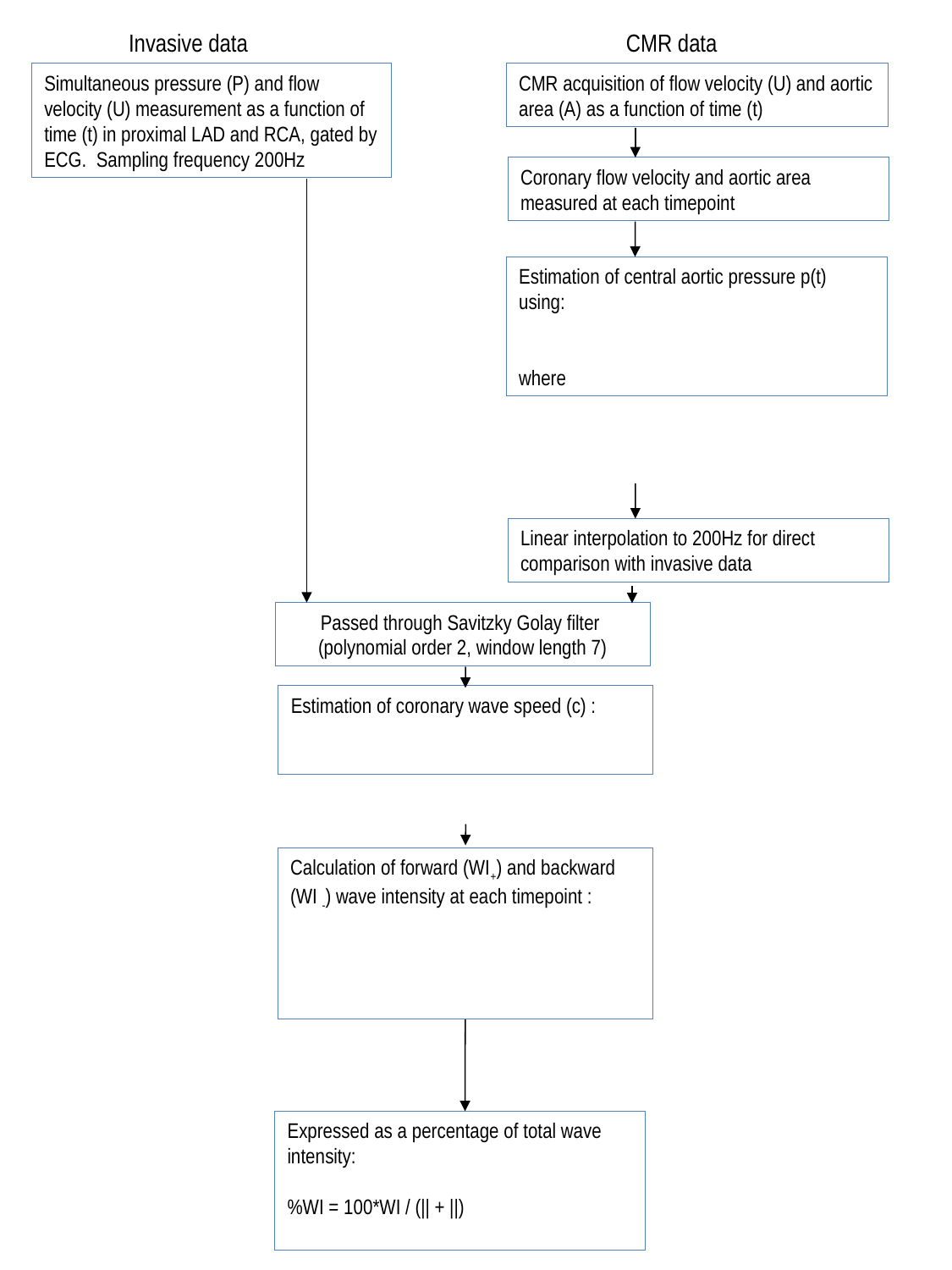

CMR data
Invasive data
Simultaneous pressure (P) and flow velocity (U) measurement as a function of time (t) in proximal LAD and RCA, gated by ECG. Sampling frequency 200Hz
CMR acquisition of flow velocity (U) and aortic area (A) as a function of time (t)
Coronary flow velocity and aortic area measured at each timepoint
Linear interpolation to 200Hz for direct comparison with invasive data
Passed through Savitzky Golay filter
(polynomial order 2, window length 7)
