# Supplementary material for: Feasibility of cardiovascular magnetic resonance derived coronary wave intensity analysis
Source: J Cardiovasc Magn Reson. 2016 Dec 9;18:93. doi: 10.1186/s12968-016-0312-8 (PMC5154155; doi:10.1186/s12968-016-0312-8)
Supplement: Supplementary file 2 — Comparison of invasive and CMR-derived pressure readings. (PPTX 205 kb) [file 12968_2016_312_MOESM2_ESM.pptx]

## Slide 1
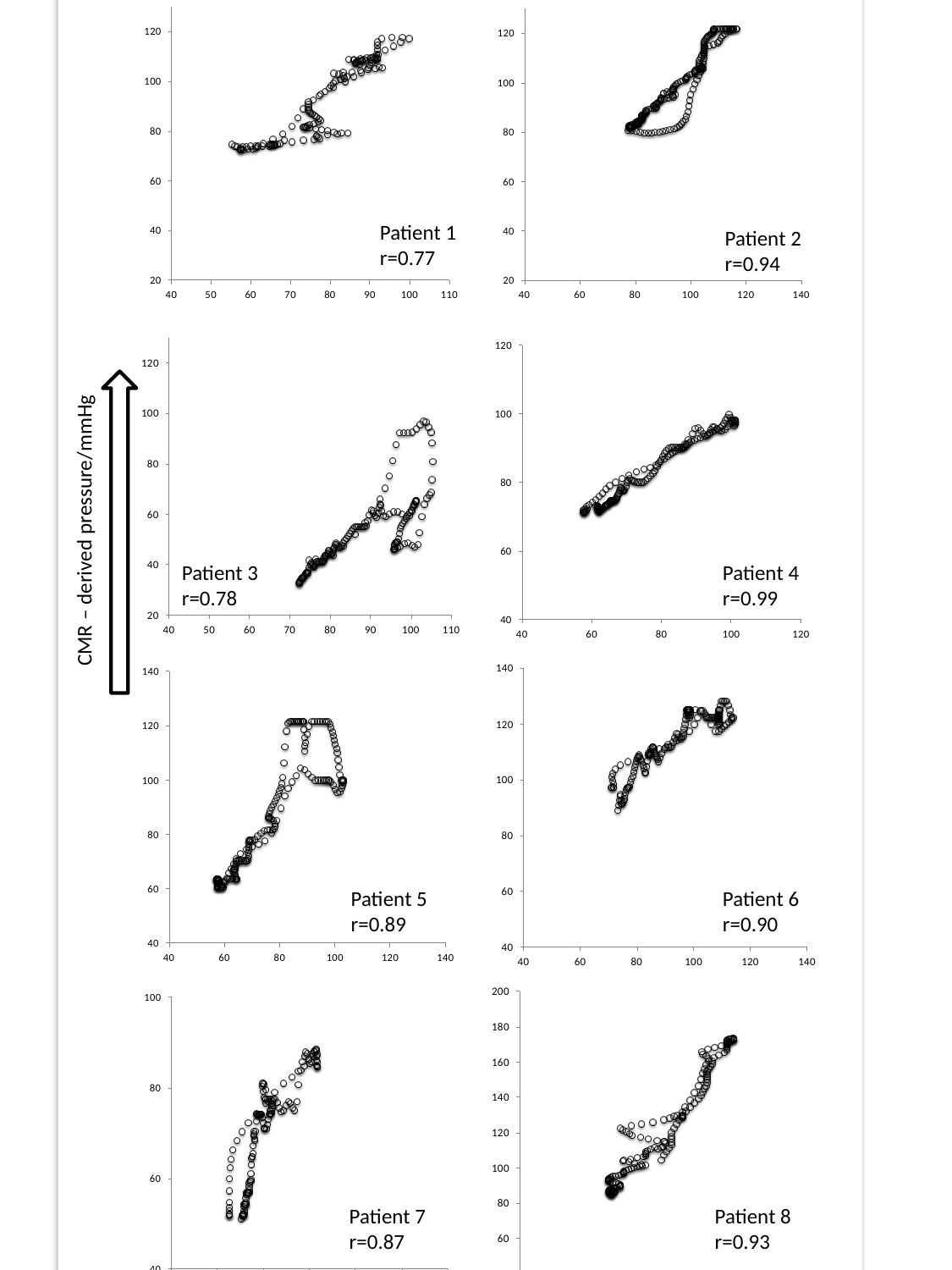

Patient 1
r=0.77
Patient 2
r=0.94
Patient 4
r=0.99
Patient 3
r=0.78
Patient 5
r=0.89
Patient 6
r=0.90
Patient 7
r=0.87
Patient 8
r=0.93
CMR – derived pressure/mmHg
Invasive pressure/mmHg
