# Supplementary material for: Feasibility of cardiovascular magnetic resonance derived coronary wave intensity analysis
Source: J Cardiovasc Magn Reson. 2016 Dec 9;18:93. doi: 10.1186/s12968-016-0312-8 (PMC5154155; doi:10.1186/s12968-016-0312-8)
Supplement: Supplementary file 3 — Comparison of invasive and CMR flow velocity readings. (PPTX 115 kb) [file 12968_2016_312_MOESM3_ESM.pptx]

## Slide 1
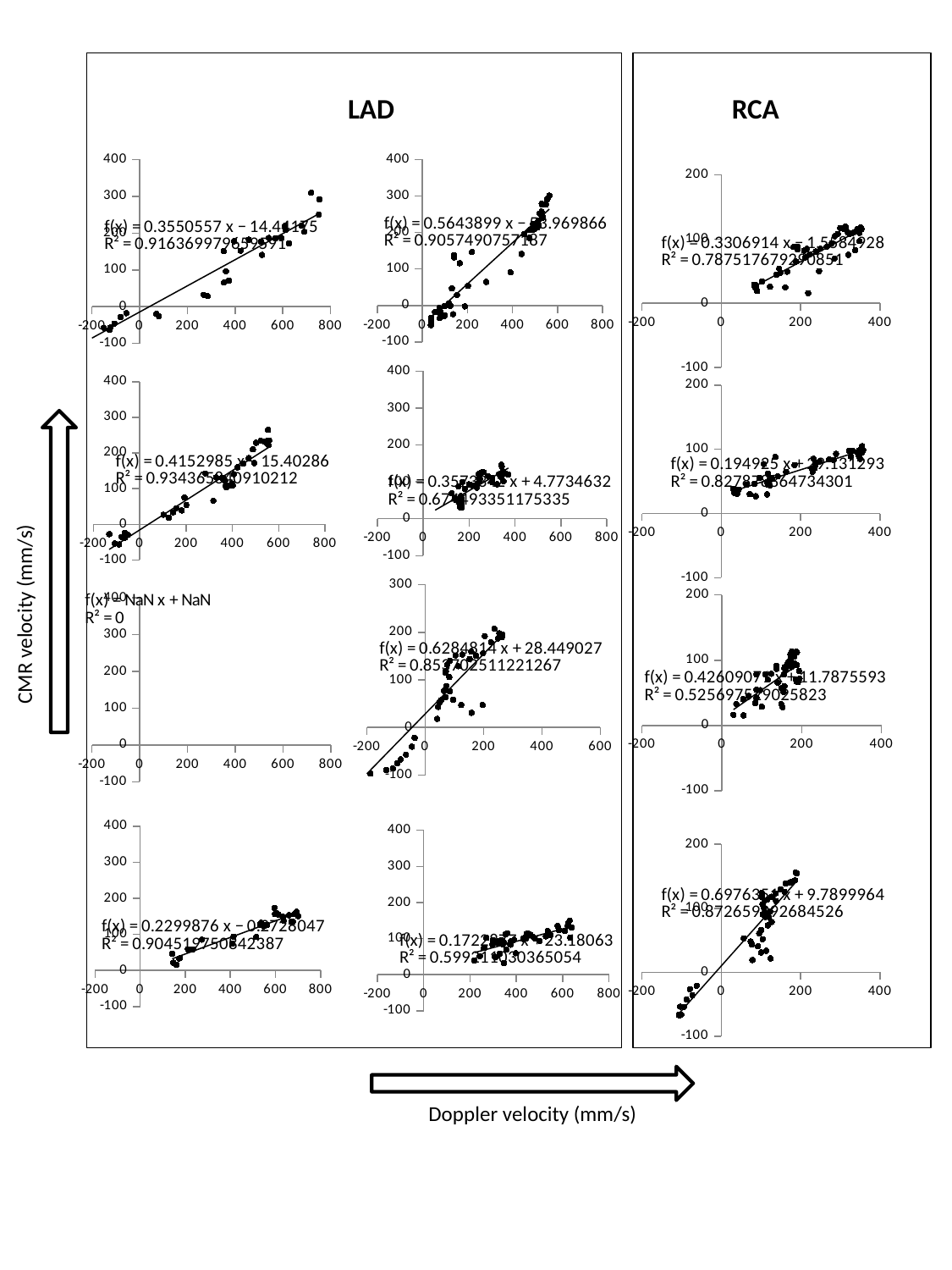

LAD
RCA
### Chart
| Category | |
|---|---|
### Chart
| Category | |
|---|---|
### Chart
| Category | |
|---|---|
### Chart
| Category | 170.0553333 150.94 |
|---|---|
### Chart
| Category | |
|---|---|
### Chart
| Category | |
|---|---|CMR velocity (mm/s)
### Chart
| Category | |
|---|---|
### Chart
| Category | |
|---|---|
### Chart
| Category | |
|---|---|
### Chart
| Category | |
|---|---|
### Chart
| Category | |
|---|---|
### Chart
| Category | |
|---|---|
Doppler velocity (mm/s)
