# Supplementary material for: Feasibility of cardiovascular magnetic resonance derived coronary wave intensity analysis
Source: J Cardiovasc Magn Reson. 2016 Dec 9;18:93. doi: 10.1186/s12968-016-0312-8 (PMC5154155; doi:10.1186/s12968-016-0312-8)
Supplement: Supplementary file 4 — Comparison of reproducibility of invasive and CMR data. The top row shows invasive vs invasive data, the middle row CMR vs CMR data and the bottom row invasive vs CMR data. (PPTX 164 kb) [file 12968_2016_312_MOESM4_ESM.pptx]

## Slide 1
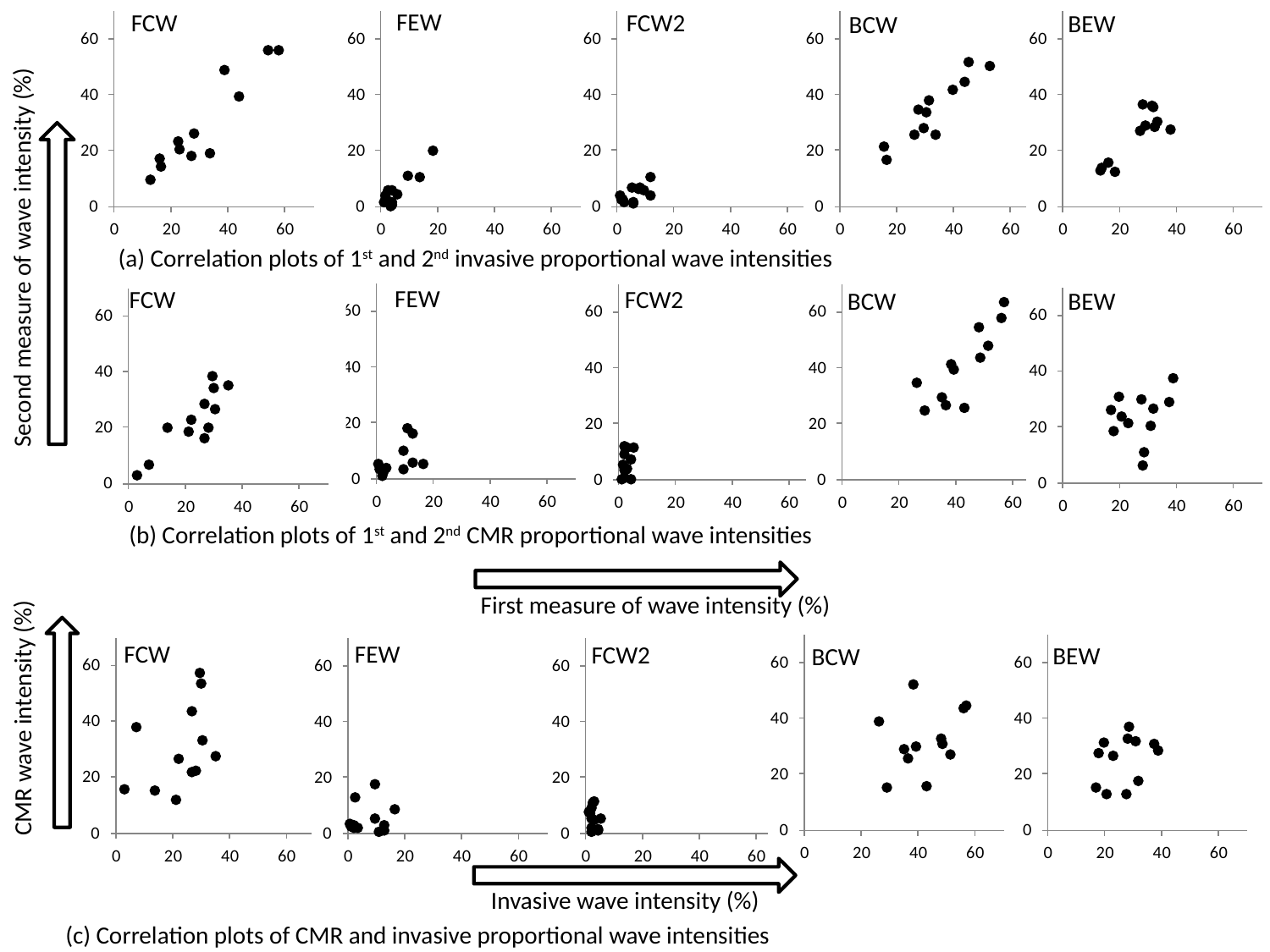

FEW
FCW
FCW2
BEW
BCW
FEW
FCW
FCW2
BEW
BCW
(a) Correlation plots of 1st and 2nd invasive proportional wave intensities
Second measure of wave intensity (%)
FEW
FCW
FCW2
BEW
BCW
(b) Correlation plots of 1st and 2nd CMR proportional wave intensities
First measure of wave intensity (%)
FEW
FCW
FCW2
BEW
BCW
CMR wave intensity (%)
Invasive wave intensity (%)
(c) Correlation plots of CMR and invasive proportional wave intensities
