# Supplementary material for: Feasibility of cardiovascular magnetic resonance derived coronary wave intensity analysis
Source: J Cardiovasc Magn Reson. 2016 Dec 9;18:93. doi: 10.1186/s12968-016-0312-8 (PMC5154155; doi:10.1186/s12968-016-0312-8)
Supplement: Supplementary file 5 — Bland Altman plots for reproducibility of individual waves using invasive (top row) and CMR (middle row) modalities. Comparison of individual waves using CMR compared to invasive data acquisition are presented in the bottom row. (PPTX 222 kb) [file 12968_2016_312_MOESM5_ESM.pptx]

## Slide 1
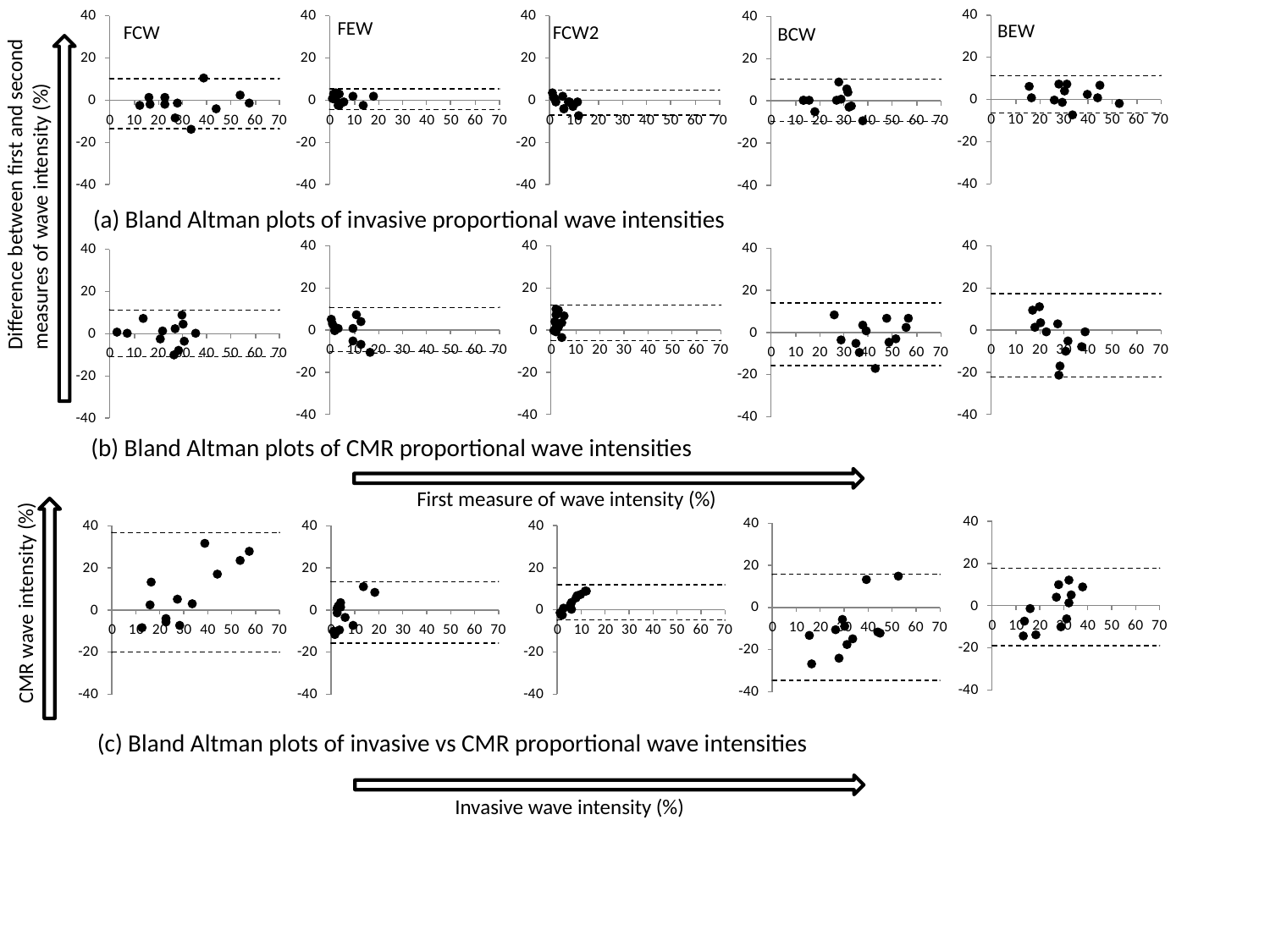

FEW
BEW
FCW
FCW2
BCW
Difference between first and second measures of wave intensity (%)
(a) Bland Altman plots of invasive proportional wave intensities
#
(b) Bland Altman plots of CMR proportional wave intensities
First measure of wave intensity (%)
CMR wave intensity (%)
(c) Bland Altman plots of invasive vs CMR proportional wave intensities
Invasive wave intensity (%)
